# Comparing Microbiome Sampling Methods in a Wild Mammal: Fecal and Intestinal Samples Record Different Signals of Host Ecology, Evolution

**DOI:** 10.3389/fmicb.2018.00803

**Published:** 2018-05-01

**Authors:** Melissa R. Ingala, Nancy B. Simmons, Claudia Wultsch, Konstantinos Krampis, Kelly A. Speer, Susan L. Perkins

**Affiliations:** ^1^The Richard Gilder Graduate School, American Museum of Natural History, New York, NY, United States; ^2^Department of Mammalogy, American Museum of Natural History, New York, NY, United States; ^3^Division of Invertebrate Zoology, American Museum of Natural History, New York, NY, United States; ^4^Sackler Institute for Comparative Genomics, American Museum of Natural History, New York, NY, United States; ^5^Department of Biological Sciences, Hunter College, City University of New York, New York, NY, United States; ^6^Center for Translational and Basic Research, Hunter College, New York, NY, United States; ^7^Institute of Computational Biomedicine, Weill Cornell Medical College, New York, NY, United States

**Keywords:** 16S rRNA, microbiome, field sampling methods, metagenomics, Chiroptera

## Abstract

The gut microbiome is a community of host-associated symbiotic microbes that fulfills multiple key roles in host metabolism, immune function, and tissue development. Given the ability of the microbiome to impact host fitness, there is increasing interest in studying the microbiome of wild animals to better understand these communities in the context of host ecology and evolution. Human microbiome research protocols are well established, but wildlife microbiome research is still a developing field. Currently, there is no standardized set of best practices guiding the collection of microbiome samples from wildlife. Gut microflora are typically sampled either by fecal collection, rectal swabbing, or by destructively sampling the intestinal contents of the host animal. Studies rarely include more than one sampling technique and no comparison of these methods currently exists for a wild mammal. Although some studies have hypothesized that the fecal microbiome is a nested subset of the intestinal microbiome, this hypothesis has not been formally tested. To address these issues, we examined guano (feces) and distal intestinal mucosa from 19 species of free-ranging bats from Lamanai, Belize, using 16S rRNA amplicon sequencing to compare microbial communities across sample types. We found that the diversity and composition of intestine and guano samples differed substantially. In addition, we conclude that signatures of host evolution are retained by studying gut microbiomes based on mucosal tissue samples, but not fecal samples. Conversely, fecal samples retained more signal of host diet than intestinal samples. These results suggest that fecal and intestinal sampling methods are not interchangeable, and that these two microbiotas record different information about the host from which they are isolated.

## Introduction

The vertebrate bacterial gut microbiome is intimately linked to host physiology, nutrition, and health (e.g., Hooper et al., 2012; Sommer and Bäckhed, 2013; The Human Microbiome Project Consortium, 2013; Mosca et al., 2016). The advent of culture-free metagenomic techniques has created a microbial ecology revolution wherein researchers can easily assess the composition, diversity, and structure of microbes in relation to their host animals (Gilbert et al., 2014). While most microbiome research has focused on humans and model organisms, microbiome studies in wildlife have recently gained popularity in light of the potential power of the microbiome to shape host evolution and ecology (e.g., Ley et al., 2008; Amato, 2013; Hird, 2017). In order to draw biologically meaningful inferences across such studies, it is essential to develop protocols that minimize bias and artifacts in samples collected from wild organisms. Many studies have compared the effects of sample preservation media, DNA extraction kits, sequencing platform, and bioinformatics pipelines on microbiome community analysis (Dominianni et al., 2014; Choo et al., 2015; Fouhy et al., 2016; Glassing et al., 2016; Song et al., 2016; Vandeputte et al., 2017), but all of these steps take place after sample collection is complete. No study currently addresses whether different collection methods have similar impacts on downstream microbial community analyses in wild mammals.

Many studies of wild mammal microbiomes have sampled feces as a proxy for the gut microbiome (Schwab et al., 2009; Amato, 2013; Amato et al., 2014; Kohl et al., 2015; Menke et al., 2015; Phillips et al., 2017). Fecal collection is a convenient method to examine the gut microbiome because it is relatively non-invasive and allows for repeated sampling of individuals through time. However, fecal microbiomes can be compromised if contamination occurs or time elapses between sample deposition and collection. A recent study demonstrated that microbial communities in springbok and giraffe fecal samples left at ambient temperature shifted considerably over the course of a week following defecation (Menke et al., 2015), suggesting that fecal collection may be better suited to studies that can ensure rapid collection and preservation of fecal samples.

As an alternative to fecal sampling, other studies focused on non-human subjects have destructively sampled the host intestinal mucosa to retrieve a microbiome sample (Phillips et al., 2012; Carrillo-Araujo et al., 2015; Hird et al., 2015). This method of collection circumvents the issue of community shifts associated with fecal samples, because intestinal sections are retrieved immediately from euthanized animals and preserved prior to microbiome analysis. However, this collection method is not ideally suited to all sampling schemes; it is impossible for studies that require repeated sampling of the same individual and it is particularly poorly suited for expansive sampling because conservation considerations or permitting constraints may preclude the sacrifice of numerous animals (Carrillo-Araujo et al., 2015). Furthermore, this method of collection is not an option for studies focusing on rare or endangered hosts. To circumvent this issue, some studies have employed rectal swabbing to sample intestinal microbiota without sacrificing the animal (Alfano et al., 2015), but this method is less commonly used on small animals, presumably due to the increased risk of injury to a small animal's rectum during swabbing. In sum, all collection and sampling methods have practical benefits and limitations that must be considered before a method is chosen.

A question central to choosing between fecal and intestinal sampling methods is a deceptively simple one: how different are the bacterial gut communities recovered from these sample types? Several studies from the human microbiome literature comparing fecal collection and rectal biopsies suggest that the intestinal lumen and mucosa may be colonized by distinct microbial communities (Durbán et al., 2011; Araújo-Pérez et al., 2012; Tang et al., 2015; Yasuda et al., 2015). Differences between these communities may be reflective of different physiological processes occurring in the intestinal lumen versus the mucosa (Tang et al., 2015). Bacteria of the intestinal mucosa have been shown to directly interact with the host immune system through Toll-like receptors located in the intestinal epithelia (Igartua et al., 2017). Fecal samples may be reflective of the luminal environment, including bacteria ingested with food, whereas direct sampling of the intestinal mucosa may be more representative of an endogenous microbiome co-evolving with the host. Fecal microbial communities may shift following defecation as a result of exposure to oxygen, moisture, and sunlight, which may further reduce similarity to the mucosal microbiome. As a result, meta-analyses of studies using different sampling schemes may not directly comparable, since biologically meaningful sources of variation may be obscured by variation arising from sampling methodology. Despite these considerations, the magnitude and significance of any differences between fecal and intestinal samples has not been examined outside of humans and laboratory animals under controlled circumstances. To address these questions in a wild mammal system, we sampled feces (guano) and the distal-most portion of the intestinal mucosa from 19 species of adult bats from Lamanai Archaeological Reserve near Indian Church, Belize and compared bacterial communities between sample types. We hypothesized the following: (1) that the microbial communities in guano and intestinal mucosa are distinct, and (2) that intestinal mucosa microbiomes show phylogenetic signal of host evolutionary history, while guano microbiomes would be less phylogenetically constrained.

## Materials and methods

### Field collection of fecal and intestinal tissue samples

Sampling took place at Lamanai Archaeological Reserve and Lamanai Outpost Lodge near Indian Church, Orange Walk District, Belize (17.75117 N, 88.65446 W) during the dry season, 24 April−5 May 2017 (Figure S1). All research was conducted in accordance with accepted standards for humane capture, handling, and sacrifice of bats published by the American Society of Mammalogists (Sikes et al., 2016) and approved Institutional Animal Care and Use Committee protocols (AMNH IACUC 20170403). Specimens were collected under the Belize Forestry Department Scientific Research and Collecting Permit WL/2/7/17 (21).

Bats were captured using a combination of ground-level mist nets, high nets, and harp traps. We immediately recovered captured bats from nets and placed individuals in separate clean holding bags to await processing. We identified all individuals to species following Reid (2009) and primary literature sources cited therein, and recorded sex, reproductive condition, forearm length, and body mass of each individual at the field station laboratory. We collected 0.25–0.5 mg of guano directly from each bat during handling when possible or from the bottom of the holding bag using sterile forceps. Bags were checked frequently to ensure freshness of samples. We placed fecal samples in sterile barcoded tubes filled with RNAlater. Individuals from whom guano had been collected were sacrificed, and we subsequently dissected out the distal intestine of each voucher specimen and placed each sample in RNAlater. Care was taken to avoid cross-contamination of tools and workspaces; tools used to manipulate bat tissues were twice sterilized in 10% bleach and rinsed with water between individuals. In addition, holding bags were laundered with soap in an industrial laundry between uses to minimize contamination from previous net nights. All voucher specimens were deposited in the Mammalogy collections at the American Museum of Natural History (AMNH), NY (Table 1).

**Table 1:** Summary of samples included in this study, including classification information about host diet, sex, collection locality, and sample types recovered.

**Family**	**AMNH Cat. No**.	**Field No**	**Species**	**Diet**	**Sex**	**Sub-site^a^**	**Sample type**
Emballonuridae	M-279587	MRI054	*Rhynchonycteris naso*	Insectivore	F	Lamanai	Intestine
	M-279558	MRI020	*Saccopteryx bilineata*	Insectivore	F	Lamanai	Guano
	M-279574	MRI040	*Saccopteryx bilineata*	Insectivore	M	Lamanai	Intestine, Guano
	M-279509	NPD288	*Saccopteryx bilineata*	Insectivore	F	Lamanai	Intestine
Molossidae	M-279506	NPD285	*Eumops nanus*	Insectivore	M	Savanna	Intestine
	M-279533	AMB001	*Molossus rufus*	Insectivore	F	Lamanai	Intestine, Guano
	M-279528	BPO014	*Molossus rufus*	Insectivore	M	Lamanai	Guano
	M-279555	MRI017	*Molossus rufus*	Insectivore	M	Lamanai	Guano
	M-279492	NBS1236	*Molossus rufus*	Insectivore	F	Lamanai	Intestine
Mormoopidae	M-279575	MRI041	*Pteronotus davyi*	Insectivore	F	Lamanai	Intestine
	M-279581	MRI048	*Pteronotus davyi*	Insectivore	F	Ka'kabish	Intestine
	M-279541	MRI003	*Pteronotus mesoamericanus*	Insectivore	F	Lamanai	Intestine
	M-279548	MRI010	*Pteronotus mesoamericanus*	Insectivore	F	Ka'kabish	Guano
	None	MRI015	*Pteronotus mesoamericanus*	Insectivore	F	Ka'kabish	Guano
Phyllostomidae	M-279572	MRI037	*Artibeus lituratus*	Frugivore	F	Lamanai	Intestine, Guano
	M-279576	MRI042	*Artibeus lituratus*	Frugivore	M	Lamanai	Intestine, Guano
	M-279579	MRI046	*Artibeus lituratus*	Frugivore	F	Lamanai	Intestine, Guano
	M-279545	MRI007	*Carollia sowelli*	Frugivore	F	Lamanai	Intestine
	M-279561	MRI023	*Chrotopterus auritus*	Carnivore	M	Ka'kabish	Guano
	M-279562	MRI024	*Chrotopterus auritus*	Carnivore	M	Ka'kabish	Guano
	M-279563	MRI025	*Chrotopterus auritus*	Carnivore	F	Ka'kabish	Guano
	M-279567	MRI031	*Dermanura phaeotis*	Frugivore	M	Lamanai	Intestine
	M-279569	MRI033	*Dermanura watsoni*	Frugivore	M	Lamanai	Intestine
	M-279535	AMB003	*Desmodus rotundus*	Sanguivore	F	Lamanai	Intestine
	M-279503	NPD282	*Desmodus rotundus*	Sanguivore	M	Lamanai	Guano
	M-279584	MRI051	*Glossophaga soricina*	Omnivore	M	Lamanai	Intestine
	M-279582	MRI049	*Lophostoma evotis*	Insectivore	F	Lamanai	Guano
	M-279539	MRI001	*Sturnira parvidens*	Frugivore	M	Lamanai	Intestine, Guano
	M-279549	MRI011	*Sturnira parvidens*	Frugivore	F	Ka'kabish	Intestine
	M-279525	BPO010	*Trachops cirrhosus*	Carnivore	M	Ka'kabish	Intestine
	M-279551	MRI013	*Trachops cirrhosus*	Carnivore	F	Ka'kabish	Intestine, Guano
	M-279564	MRI026	*Trachops cirrhosus*	Carnivore	F	Ka'kabish	Intestine
	M-279554	MRI016	*Uroderma bilobatum*	Frugivore	M	Savanna	Intestine
Vespertilionidae	M-279517	BPO002	*Myotis keaysi*	Insectivore	F	Lamanai	Guano
	M-279540	MRI002	*Myotis keaysi*	Insectivore	M	Lamanai	Intestine, Guano
	M-279543	MRI005	*Myotis keaysi*	Insectivore	M	Lamanai	Guano
	M-279534	AMB002	*Rhogeessa aeneus*	Insectivore	F	Lamanai	Intestine, Guano
							

*If a voucher specimen was collected, its catalog number at the American Museum of Natural History is also listed. Diet classification is based on Reid (2009) and Clare et al. (2014)*.

a *“Lamanai” refers to both Lamanai Outpost Lodge and Lamanai Archaeological Reserve. Because these sites are adjacent and fewer than 5 km apart, we group them together here for convenience. For further reference, please see Figure S1*.

### DNA extraction

All laboratory protocols were performed at the Sackler Institute for Comparative Genomics at the AMNH. We performed DNA isolations and library preparations in a UV-sterilized laminar flow hood to minimize aerosol contamination. Intestinal tissue was scraped using sterilized razor blades. Guano and intestinal scrapings were placed in bead tubes and mechanically disrupted with a Disruptor Genie (Scientific Industries, Bohemia, NY) for 45 s^−1^ min. We extracted microbial DNA from guano and intestinal mucosa samples using the MO BIO PowerLyzer™ PowerSoil® DNA Isolation kit (MO BIO Laboratories, Carlsbad, CA), using 0.25 mg of sample and following the manufacturer's instructions with the following amendment: samples were incubated at room temperature for two min on the extraction column membrane prior to final elution (QIAGEN, pers. comm.). Samples with high organic content were further purified using the PowerClean® Pro DNA Clean-Up Kit (MO BIO Laboratories, Carlsbad, CA). Each extracted DNA sample was quantified using a Qubit™ 2.0 Fluorometer and High Sensitivity dsDNA reagent kit (Invitrogen, Carlsbad, CA). A total of 55 DNA samples, 29 intestinal and 24 guano, were used for metagenomic library preparation, including extraction and PCR negative controls to account for contamination at each step in the library preparation.

### 16S amplicon library preparation

We followed the Illumina® 16S Metagenomic Library Preparation guidelines to create 16S rRNA amplicon libraries. We first amplified the hypervariable (V4) region of the 16S rRNA SSU gene from each sample using primers 515f (5′-TCGTCGGCAGCGTCAGATGTGTATAAGAGACAGGTGYCAGCMGCCGCGGTAA-3′) and a revised 806Rb (5′-GTCTCGTGGGCTCGGAGATGTGTATAAGAGACAGGGACTACNVGGGTWTCTAAT-3′) (Caporaso et al., 2011; Apprill et al., 2015) with Illumina® sequencing adaptors (Illumina Inc., San Diego, CA). For each of the 55 samples, a total of three amplicon PCR replicates were performed to control for PCR bias, which typically occurs in the first few rounds of replication (Suzuki and Giovannoni, 1996; Polz and Cavanaugh, 1998). Briefly, the initial amplicon PCR was performed in 25 μL reactions using 2.5 μL of input DNA, 1.0 μM forward and reverse primers, as well as 12.5 μl of KAPA Taq HiFi HotStart High Fidelity ready mix (KAPA Biosystems, Woburn, MA). Cycling conditions were as follows for the amplicon PCR: an initial denaturation at 95°C for 3 min, followed by 25 cycles of 95°C (30 s), 55°C (30 s), and 72°C (30 s), with a final extension at 72°C for 5 min. Following the PCR, triplicate amplicon reactions originating from the same DNA sample were pooled and cleaned using AMPure® XP beads (Agencourt Biosciences, Beverly, MA). We checked the library size for a subset of samples using a BioAnalyzer 1,000 chip (Agilent Technologies, Palo Alto, CA) and then performed an indexing PCR using Nextera XT Index Primer Set A (Illumina, Inc., San Diego, CA) in 50 μL reactions. Cycling conditions for the indexing PCR were 95°C for 3 min, followed by eight cycles of 95°C (30 s), 55°C (30s), and 72°C (30 s) with a final extension at 72°C for 5 min. We checked the size and quality of a representative subsample of the indexed libraries using a BioAnalyzer 1,000 chip and performed a final AMPure® cleanup. Libraries were quantified, normalized to 4 nM, pooled, and then sequenced at the Bioinformatics Core Infrastructure Laboratory (BCIL) at the City University of New York using an Illumina MiSeq platform (Illumina, Inc., San Diego, CA) targeting 2 × 300 bp paired-end sequence reads.

### Data analysis

The sequencing run produced a total of 4,336,591 raw reads across our 55 input libraries. We analyzed these metagenomic data using the open-source QIIME2 pipeline (Caporaso et al., 2010; Kuczynski et al., 2012). We first quality filtered sequences using the DADA2 algorithm (Callahan et al., 2016) as a QIIME2 plugin. DADA2 joins paired-end reads together, and then implements a quality-aware correcting model for amplicon data that denoises, removes chimeras and residual PhiX reads, dereplicates DNA reads, and calls amplicon sequence variants (ASVs). ASV generation was recently shown to outperform Operational Taxonomic Unit (OTU) clustering, resulting in fewer spurious reads (Callahan et al., 2017). Unlike OTU clustering, ASVs are not compared to a reference database and retained when they meet an arbitrary similarity cutoff (usually 97%). As a result, novel bacterial taxa from wildlife microbiomes, which may be underrepresented in 16S databases, are not discarded as potential sequencing artifacts simply because they have no close relative in the database (Callahan et al., 2017). We trimmed the first 35 bases of each read and truncated sequences to 187 bp for DADA2 analysis based on average quality scores determined for both forward and reverse 300 bp reads. After quality filtering, the dataset contained 1,434,316 reads across 55 samples, with an average of 26,000 sequences per sample. We conducted a masked alignment using MAFFT (Katoh et al., 2002) and constructed a phylogeny from these sequences using the QIIME2 FastTree plugin (Price et al., 2010). Using rarefaction, we chose a subsampling depth of 1,000 sequences per sample, which gave us a final rarefied dataset of 46 samples (Figure S3). Seven samples and both the negative extraction control and negative PCR control contained fewer than 1,000 sequences and were dropped from further analysis. 16S sequences were assigned to taxonomic groups using the Greengenes database as a reference (DeSantis et al., 2006). In QIIME2, we also filtered the feature table to remove sequences classified as mitochondria or chloroplasts, as these are common non-target amplicons in microbiome studies (de la Cuesta-Zuluaga and Escobar, 2016).

We computed two alpha diversity metrics to measure the richness of the communities within samples: the Shannon Index (Shannon, 1948), which is a richness metric derived from information theory that is sensitive to community evenness, and Faith's Phylogenetic Diversity index (Faith, 1992), which considers the phylogenetic relatedness of taxa in each sample by computing the sum of all branch lengths across the minimum spanning path. Shannon richness was computed using the “estimate_richness” function in R package *phyloseq* v. 1.22.3 (McMurdie and Holmes, 2013), while Faith's Phylogenetic Diversity and rarefaction curves (Figure S2) were computed directly using QIIME2. Because some of our samples were related by being isolated from the same host, we compared richness and diversity of sample types using the non-parametric Wilcoxon sign rank test as implemented by the base R function “wilcox.test.” We used permutational multivariate analysis of variance, or perMANOVA, (Anderson, 2001) on both weighted and unweighted Unifrac (Lozupone et al., 2006) distance matrices to test for differences in microbiome beta diversity between sample types, and tested for homogeneity of variance among samples using “betadisper” as implemented in the R package *vegan* (Oksanen et al., 2017). We visualized relative abundances of bacteria in paired sample types using the “plot_heatmap” function in *phyloseq* v. 1.22.3, and visualized ordination plots using Principle Coordinate Analysis (PCoA). Finally, we used R packages *vegan* v. 2.4-5 (Dixon, 2003) and *phytools* v. 0.6-44 (Revell, 2012) to test for phylogenetic congruence between the host phylogeny and microbiome structure, using the Robinson-Foulds metric to assess topological similarity (Robinson and Foulds, 1981).

## Results

### Bat species sampled

We captured 17 species of bats distributed across five families. Our dataset contains representatives of diverse feeding ecologies, including obligate blood feeders, strict frugivores, and omnivores with complex diets of plant, insect, and/or vertebrate material (Table 1). Across our 55 sequenced libraries, our dataset included 37 individual bats, with paired data (fecal and intestinal samples) for 9 individuals.

### Microbiome composition and diversity of neotropical bats

**Figure 1:**
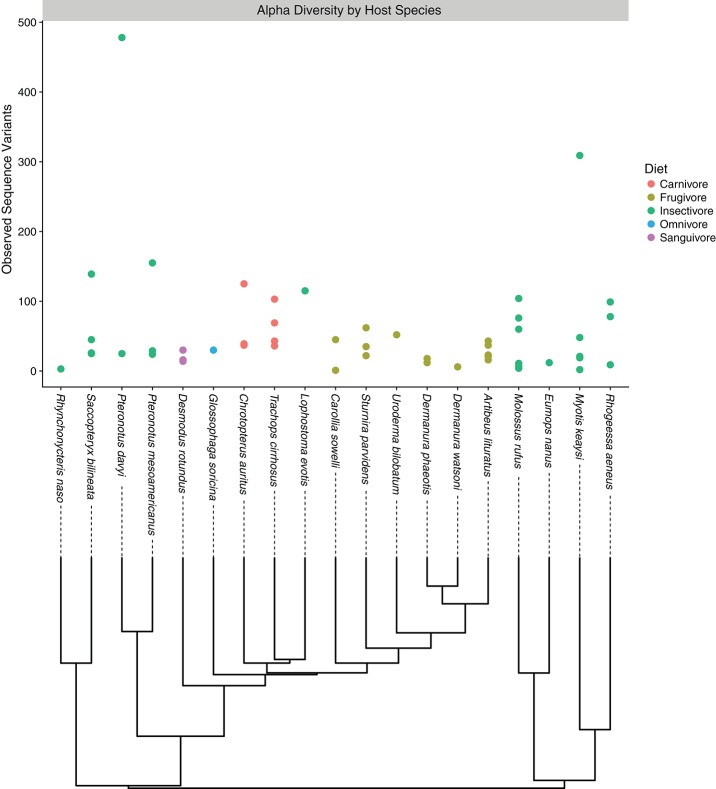
Alpha diversity plot depicting the number of observed sequence variants associated with each host bat species. Each point corresponds to an individual sample, while colors correspond to the feeding guild to which the host bat belongs. Under the horizontal axis, evolutionary relationships among host species are depicted.

Among all samples, only a handful of bacterial phyla dominated community compositions; most abundant were the Proteobacteria, Firmicutes, and Tenericutes; the Actinobacteria, Bacteroidetes, and Synergistes were also present to a lesser degree (Figure S2). When considering different sample types, Tenericutes tended to be more abundant in intestinal samples than guano samples. Conversely, Bacteroidetes were more common in guano samples than in intestinal samples (Figure S2). The overall profile of bacterial phyla we recovered was consistent with previous reports from Neotropical bats (Carrillo-Araujo et al., 2015; Nishida and Ochman, 2017). We summarized alpha diversity among the focal bat taxa by comparing the number of observed sequence variants for each species. Alpha diversity of microbiome communities varied considerably within and among host species; *Pteronotus davyi* had highest number of observed ASVs (478), while *Rhynchonycteris naso* had the most depauperate microbial community, consisting of only three ASVs. Across dietary classes, frugivores and the lone sanguivore, *Desmodus rotundus*, had relatively low diversity. Omnivores had intermediate diversity, while insectivorous bat species seem to have high microbial diversity (Figure 1). These results are consistent with a previous study, which found insectivorous bat species to have the most diverse microbiomes; within the phyllostomids, the same study found *D. rotundus* to have the most depauperate microbiome, with insect eating and plant visiting species to be slightly more diverse (Phillips et al., 2012).

### Guano and intestinal mucosa are compositionally distinct

**Figure 2:**
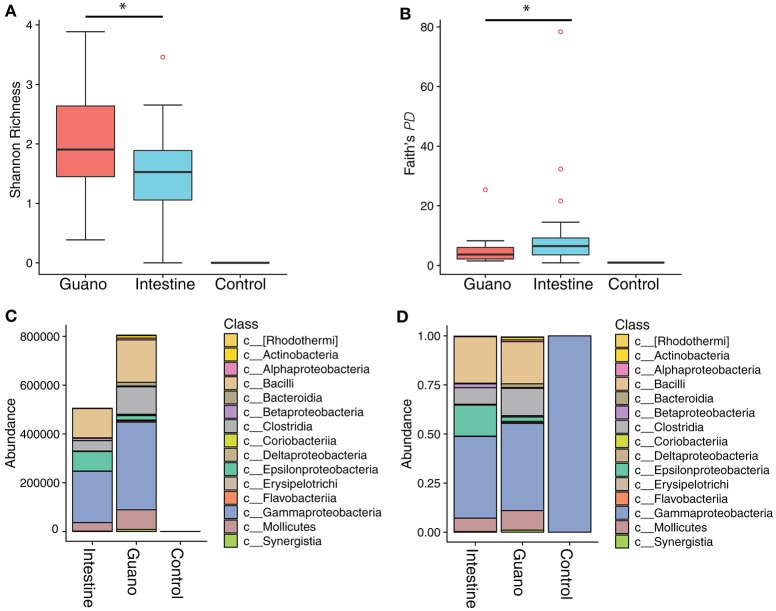
Alpha diversity box plots showing **(A)** Shannon richness and **(B)** Faith's phylogenetic diversity of microbial communities sampled two ways from Neotropical bats. An extraction negative and PCR negative were also sequenced to control for contamination. The absolute abundance of the top 20 most abundant classes in each sample are shown in panel **(C)**. Panel **(D)** shows the relative abundance of the same top 20 classes. Note that the negative control maps to a single gammaproteobacterial lineage in the genus *Cellvibrio*. *Denotes significant difference at the *P* ≤ 0.05 level.

We characterized differences in bacterial community composition between guano samples versus and samples. We found that Shannon richness was higher in guano samples compared to intestine (${\bar{H}}_{\text{guano}}$ = 2.05, ${\bar{H}}_{\text{intestine}}$ = 1.49, paired Wilcoxon signed rank *W* = 106, *P* = 0.02) (Figures 2A,B). However, phylogenetic diversity was higher in intestinal samples than guano samples (${\bar{D}}_{\text{guano}}$ = 4.86, ${\bar{D}}_{\text{intestine}}$ = 10.04, Wilcoxon signed rank *W* = 110, *P* = 0.02). Both communities were largely dominated by similar bacterial taxa, with the most abundant ASVs falling into the classes Bacilli, Clostridia, Epsilonproteobacteria, Gammaproteobacteria, and Mollicutes (Figure 2C,D). However, relative and absolute abundances of the most abundant sequence variants were slightly different between sample types, with Epsilonproteobacteria represented in higher proportion in intestine than guano (Figure 2D). We identified only one ASV in our extraction negative control; this sequence was a 100% match to the gammaproteobacterial lineage *Cellvibrio* (Figure 2D). This bacterium was likely a contaminant introduced by the extraction kit, as it was not present in meaningful abundance in test samples, so we filtered this taxon from further analyses.

**Figure 3:**
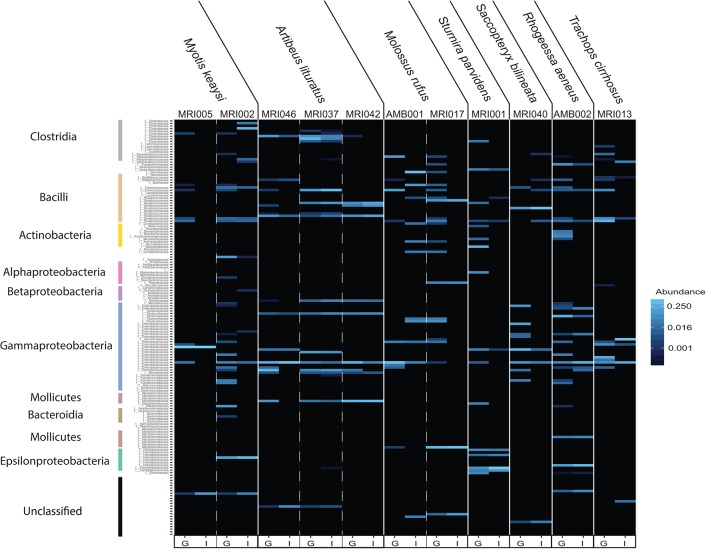
Heat map depicting differences in estimated bacterial family abundance between paired guano and intestinal samples collected from 7 bat species. Bacterial families are shown vertically, with colored bars corresponding to the classes shown in Figures 2C,D. Unassigned identities indicate that the sequence could not be matched with certainty to a known bacterial class and may represent novel taxa. Host individual and species are located along the top and sample type, indicated as guano (G) or intestine (I), is located along the bottom.

We next sought to examine the amount of overlap between guano and intestinal communities by displaying the community compositions as a heat map (Figure 3). The “plot_heatmap” function organizes the heat map using a user-specified ordination method (in our case, weighted Unifrac) to produce a highly interpretable order of elements. As is typical of microbiome datasets (Kurtz et al., 2015), our taxon presence matrices were sparse with only a subset of the total number of bacterial taxa representing the majority of relative abundances. The majority of the heat map therefore appears black because most taxa are low-abundance or absent from the matrix. Only those bacterial families that are more abundant than background are shown in increasing intensities of blue. We found that guano samples tended to have more abundant bacterial families within each Class than did intestinal samples (Figure 3). When a bacterial taxon was present in both guano and intestine, there were pronounced differences in abundance of the shared taxon between the two sample types (Figure 3). Interestingly, potentially novel bacterial taxa tended to be either completely absent or markedly less abundant in guano samples compared to intestinal samples (Figure 3, “unassigned”). In bat species for which we had paired intestinal and guano samples from the same individuals (*n* = 11), we found species-specific patterns in the most abundant bacterial taxa, but also a degree of intra-specific variation in the presence and abundance of some taxa. For example, all *Artibeus lituratus* (the Great Fruit-eating Bat) samples, regardless of sample type, contained members of the Enterococcaceae and Streptococcaceae (Class: Bacilli), but only the intestinal sample from individual MRI037 of that species contained meaningful abundances of Pseudomonadaceae (Class: Gammaproteobacteria) and Helicobacteraceae (Class: Epsilonproteobacteria) (Figure 3).

**Figure 4:**
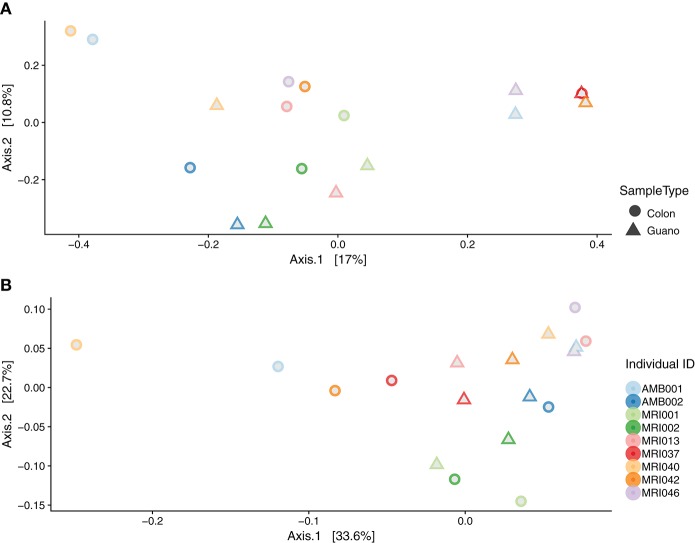
Beta diversity by sample type: principal coordinate analysis plots of **(A)** unweighted and **(B)** weighted Unifrac distances of paired intestinal and guano samples. Field numbers represent individual bats whose species descriptions are as follows: *Artibeus lituratus* (MRI037, MRI042, MRI046), *Molossus rufus* (AMB001, MRI017), *Myotis keaysi* (MRI005, MRI002), *Rhogheessa aeneus* (MRI002), *Saccopteryx bilineata* (MRI040), *Sturnira parvidens* (MRI001), and *Trachops cirrhosus* (MRI013).

We used perMANOVA on weighted and unweighted Unifrac distances computed from the rarefied dataset (*n* = 46) to test for dissimilarities in community composition among intestinal and guano samples. perMANOVA analysis performed on unweighted Unifrac distances showed that intestinal and guano samples clustered by sample type (*P* = 0.001, *F* = 3.354, *r*^2^ = 0.072). However, a multivariate test for homogeneity of variance was significant (permutations = 999, *P* = 0.014, *F* = 5.937), suggesting that differences in these communities could be due to differences in within-group dispersions rather than differences in centroid position (Figure S4). We suspected that the differences in dispersion might be due to high inter-individual variation in community structure, so we repeated the analysis only using paired samples (i.e., guano and intestinal communities sampled from the same individuals, *n* = 22). In the paired test, we found that distances estimated with unweighted Unifrac (*P* = 0.048, *F* = 1.879, *r*^2^ = 0.11) were significantly different between sample types, and homogeneity of dispersion was not rejected (*P* = 0.15), suggesting that the microbial communities in intestine and guano are indeed different when inter-individual variation is controlled (Figure 4A). Weighted Unifrac distances were also significantly different between sample types (*P* = 0.046, *F* = 2.07, *r*^2^ = 0.103, but homogeneity of dispersion was rejected (*P* = 0.023), suggesting that abundance-weighted differences may be driven by differences in within-group dispersions (Figure 4B).

### Intestinal samples reflect host phylogeny, while fecal samples reflect diet

**Figure 5:**
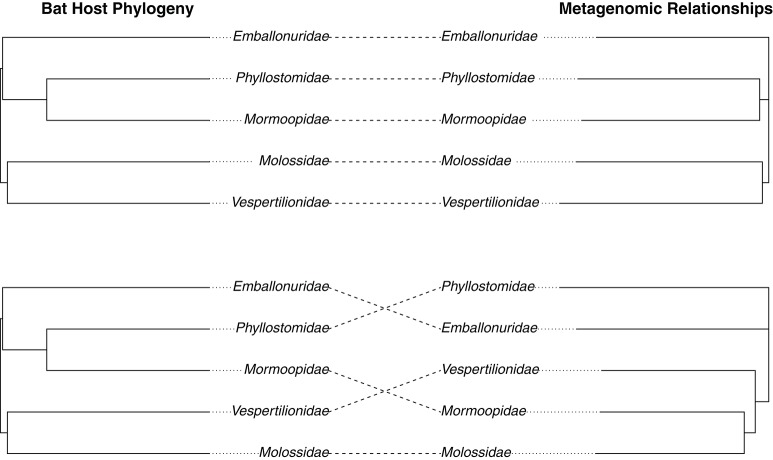
Congruence between host bat phylogeny and microbiome relationships based on **(A)** intestinal and **(B)** guano bacterial communities. Metagenomic relationships were inferred by computing unweighted Unifrac distance matrices for each sample type merged according to host family. Distance matrices were used to create neighbor-joining trees inferring metagenomic relationships. The host phylogeny was taken from a Bayesian analysis of bat speciation rates by Shi and Rabosky (2015) and collapsed to family level using a custom script. Topological similarity between metagenomic and host trees was assessed using the Robinson-Foulds metric.

We hypothesized that the intestinal microbiome would be more reflective of host evolutionary history since mucosal bacteria are more closely associated with the host than luminal or fecal bacteria and as such would be predicted to show stronger coevolutionary patterns. To test for signatures of host phylogeny on metagenomic community composition, we first merged communities of each sample type to the level of host family using function “merge_samples” in the *phyloseq*. A previous study showed that detectable influences of host evolution are most often recovered in ancient nodes on the host phylogeny, such as host family, because variation among individuals can obscure patterns at the level of host species (Phillips et al., 2012); this, in conjunction with empirical evidence for intraspecific variation in community structure (Figures 1, 3), led us to believe this phylogenetic scale would be appropriate. We next constructed unweighted Unifrac distance matrices for each merged sample type, and used these distance matrices to construct neighbor-joining trees. The resulting phylogenies were compared to a phylogeny of host bat families from Shi and Rabosky (2015) to assess phylogenetic congruence. We found that intestinal microbiota produced a phylogeny that was nearly identical to the phylogeny of the hosts (Robinson-Foulds distance = 0.00), while guano microbiota produced an incongruent phylogeny that inferred strictly insectivorous families as being more closely related (Robinson-Foulds distance = 4.0) (Figure 5). Given the fact that guano microbiomes grouped bat families with similar feeding habits together, we performed PERMANOVA on unweighted Unifrac distances from all guano samples to test for differences in beta diversity among diet groups. We found significant differences in microbiome community structure among bats of various feeding guilds (*P* = 0.007, *F* = 1.61, *r*^2^ = 0.21). Pairwise comparisons between groups revealed strong differences in community structure between carnivorous and frugivorous bats, and also between insectivores compared with frugivores and sanguivores (Table 2). Pairwise comparisons between the other groups were not significantly different, likely owing to the limited sample size within sanguivores (*n* = 2) and carnivores (*n* = 4).

**Table 2:** Pairwise beta-diversity comparisons of guano from bats of different feeding guilds.

**Group 1**	**Group 2**	**Sample size**	**Permutations**	**pseudo-F**	***p*-value**
Carnivore	Frugivore	9	999	1.90	0.031^**^
	Insectivore	16	999	1.43	0.058
	Sanguivore	6	999	1.66	0.057
Frugivore	Insectivore	17	999	1.88	0.012^**^
	Sanguivore	7	999	2.10	0.064
Insectivore	Sanguivore	14	999	1.61	0.049^**^

** *P ≤ 0.05*.

## Discussion

Understanding the gut microbiome and its contributions to host fitness and evolution is an increasingly important goal for evolutionary biology, ecology, and conservation (Amato, 2013; Hird, 2017; Kohl, 2017), but the study of microbiomes is complicated by numerous sources of bias that can impact any step of the pipeline (Kim et al., 2017). While sources of bias in the preservation, sequencing, and analysis phases of microbiome studies are relatively well documented, the impact of methods used to sample these communities in the first place has remained understudied. Here, we report on the microbiome composition of a diverse assemblage of Neotropical bats, and compare two common field sampling techniques to assess how sampling method impacts microbiome inference. We found that there were compositional differences between fecal and intestinal samples that could not be attributed solely to inter-individual variation in microbial community structure. We also report differences in relative abundances of shared bacterial taxa between these two sample types. Perhaps most importantly, we found that of these two sample types, the intestinal microbiota is more phylogenetically constrained than the guano microbiota. These findings have important implications for future wildlife microbiome studies whose goals are to utilize host-associated microbial communities to understand host ecology and evolution.

We documented similar patterns of taxonomic composition as have been previously reported in Neotropical bats (Phillips et al., 2012; Carrillo-Araujo et al., 2015; Nishida and Ochman, 2017) (Figure S2). In addition, we found considerable levels of inter- and intra- specific variation in microbiome composition, which is consistent with previous reports in phyllostomids (Carrillo-Araujo et al., 2015). Previous studies suggested that most mammals of the same species share a “core microbiome,” or a minimal set of shared bacterial taxa associated with a particular body site (Huse et al., 2012; Falony et al., 2016). Apart from the “core” bacteria, variation in microbiome structure within mammals of the same species has been reported in humans (Arumugam et al., 2013), koalas (Alfano et al., 2015), and *Peromyscus* mice (Baxter et al., 2015). The mechanisms supporting such inter-individual variation may be attributable to differences in host diet, or to genetic divergence among different host populations. For example, one study found evidence that microbiome similarities among populations of American pikas (*Ochotona princeps*) were congruent with the genetic relationships among the host populations (Kohl et al., 2017). Because there is no record of population structure for most bat species in our study area (Figure S1) and the dispersal tendencies of many of these species are unknown (Albrecht et al., 2007), we are unable to rule out host genetic divergence as a cause for the variation we observe in our bat microbiomes. However, it is also possible that diet may be driving some of this variation, as even fig specialists (*Artibeus spp*.) may discriminate among 14 different species of figs with very different nutritional profiles (Wendeln et al., 2000). In light of this evidence, our results highlight the need for future studies to link microbiome variation in wildlife with potential demographic and ecological covariates.

The differences we recovered between fecal and intestinal mucosa samples are likely reflective of the different processes that are known to occur in these microhabitats. Our results join a growing canon of work on humans, as well as laboratory and other captive animals that suggests that the luminal and mucosal microbiota are distinct communities that share some taxonomic overlap (Eckburg et al., 2006; Yasuda et al., 2015; Videvall et al., 2017). We found that the guano microflora showed higher species richness, but intestinal samples tended to have more phylogenetically diverse members (Figure 2). In our examination of paired sample types from the same individuals, we found a similar result, with the intestinal microbiome appearing to be a representative subset of the guano microbiome, with some unique taxa (Figures 3, 4). A possible explanation for this observation is that the guano microbiome retains the signature of bacteria ingested along with food items. Many studies have emphasized the power of host diet to shape the structure of the microbiota by altering the nutritional environment available to resident microbes (David et al., 2014; Carmody et al., 2015; Groussin et al., 2017), but it is also possible that bacteria ingested with food items serve as a source of inoculum to the luminal microbiome. Compared to other mammals, the digestive system of bats is characterized by rapid transit time of food, with complete passage in as little as 30 min (Klite, 1965; Tedman and Hall, 1985; Chivers and Langer, 1994). Potentially, the increased richness of guano microflora compared with the intestinal mucosa may be due to the presence of bacterial DNA retained in undigested material. This may be an important consideration for future studies, as taxonomic consistency of the fecal microbiota through time may be reduced in species whose diets vary seasonally (Amato et al., 2014; Smits et al., 2017) or geographically (Phillips et al., 2012; Moeller et al., 2013). Recently, it was shown that overlap in mammalian microbiome composition decays rapidly with increasing geographic distance between populations, indicating that dispersal limitations of bacteria promote *in-situ* diversification, resulting in potentially high levels of intraspecific microbiome variation in widely distributed species (Moeller et al., 2017).

Previous studies have demonstrated that the microbiome carries signal of the hosts' phylogeny, suggesting that microbial communities in the gut have co-evolved with their hosts (Ley et al., 2008; Sanders et al., 2015; Colston and Jackson, 2016; Moeller et al., 2016). In bats, the only studies to find such relationships have exclusively sampled intestinal contents (Phillips et al., 2012; Carrillo-Araujo et al., 2015). Here, we tested for phylogenetic congruence between host bats and their microbiota as inferred from both intestinal tissue samples and guano. Consistent with our expectations, we found that distances among intestinal microflora recapitulated current hypotheses of host phylogeny with little incongruence at the family level (Figure 5). However, the guano microflora produced a phylogeny that was highly incongruent with the phylogeny of the hosts (Figure 5).

The phylogeny constructed from guano sample distances inferred bat families with similar diets as being more closely related. Because our guano samples were very fresh (preserved within 5-30 minutes of defecation), the impact of environmental contamination on microbial community composition would be minimal, suggesting other factors must be driving this pattern. Of the bat families we sampled, members of Emballonuridae, Mormoopidae, Molossidae, and Vespertilionidae retain the ancestral Chiropteran feeding mode of strict insectivory, while members of the Phyllostomidae have radiated into other dietary niches such as frugivory, sanguivory, omnivory, and carnivory (Fenton et al., 2001; Kunz and Fenton, 2003). This may further imply that food-borne microbial DNA persists in fecal samples and suggests that the guano microbiome may reflect a strong signature of host ecology that overpowers any underlying phylogenetic signal. Indeed, we found that microbiome beta diversity was different in guano samples from bats of different feeding guilds (Table 2). Some studies have found microbiome convergence in distantly related mammals of similar feeding modes, and most of these studies sampled feces (Muegge et al., 2012; Delsuc et al., 2014). If our results are broadly applicable to all mammals, findings of microbiome convergence within dietary guilds could be due to the strong signal of host diet as an attribute of the fecal microbiota, whereas intestinal samples or rectal swabs may better reflect host phylogeny. Indeed, a recent meta-analysis utilizing fecal samples from 112 mammal species failed to find a signal of host phylogeny in members of the Chiroptera, likely owing to their exclusion of studies which sampled intestinal mucosal samples (Nishida and Ochman, 2017). Future studies should consider the possibility that different sample types record different information about the host, and where possible, make use of intestinal sampling if the goal is to analyze the microbiome in the context of host evolution. Importantly, our study did not consider the potential differences between intestinal mucosa and rectal swabs; currently, there is conflicting evidence about whether swabs are representative of the mucosal microbiome (Araújo-Pérez et al., 2012; Bassis et al., 2017), suggesting a need for an expansion of this study to include a comparison of rectal swab samples with intestinal and fecal sampling.

## Conclusions

Our results suggest that differences in sampling methodology can impact the inferences drawn from mammalian microbiomes. Because fecal and intestinal samples differ substantially, meta-analyses of studies that include different microbiome sample types may introduce enough noise to obscure biologically meaningful patterns of host evolution and ecology. We therefore caution researchers to maintain consistency in sampling methodology in order to preserve comparability across studies. We encourage future studies to implement similar tests to the ones we performed here if both sample types are collected. If collection of multiple sample types is not possible, we recommend tuning sampling methods to specific research goals; fecal samples may be ideal for studies seeking to analyze the microbiome in the context of host diet, while intestinal samples may be better suited for questions framed in the context of the evolution of the host. However, the reality is that destructive sampling is not always possible due to protections on endangered hosts, making rectal swabbing a reasonable alternative that deserves future consideration.

## Data accessibility

Raw, demultiplexed 16S sequences are publically available on the NCBI Sequence Read Archive under BioProject # PRJNA428973. QIIME2 mapping file and annotated feature table are available on Figshare under doi: 10.6084/m9.figshare.5975365.

## Author contributions

MI, NS, and SP conceived of the study design. MI, NS, and KS conducted field sampling, and MI, SP, CW, and KK performed laboratory portions of the study. MI and KS performed all analyses in consultation with CW and KK. All authors participated in drafting the manuscript.

### Conflict of interest statement

The authors declare that the research was conducted in the absence of any commercial or financial relationships that could be construed as a potential conflict of interest.
