# Supplementary material for: Comparing Microbiome Sampling Methods in a Wild Mammal: Fecal and Intestinal Samples Record Different Signals of Host Ecology, Evolution
Source: Front Microbiol. 2018 May 1;9:803. doi: 10.3389/fmicb.2018.00803 (PMC5938605; doi:10.3389/fmicb.2018.00803)
Supplement: Supplementary file 1 [file Image_1.PDF]

Supplementary Material

Comparing Microbiome Sampling Methods in a Wild Mammal: Fecal and Intestinal Samples Record Different Signals of Host Ecology, Evolution

Melissa R. Ingala*, Nancy B. Simmons, Claudia Wultsch, Konstantinos Krampis, Kelly A. Speer, and Susan L. Perkins

***Correspondence:** Corresponding Author: ingala.melissar@gmail.com

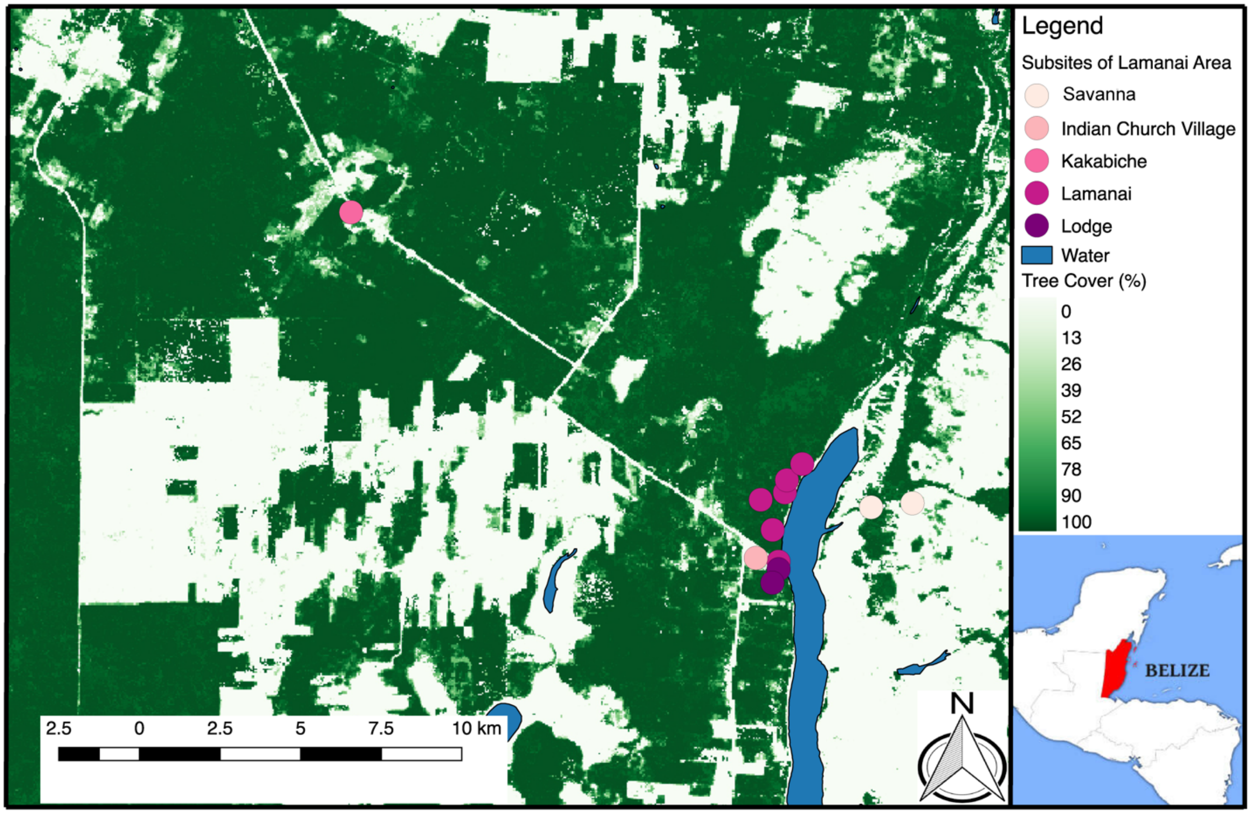

Supplementary Figure 1. Detail map of the area around Lamanai Archaeological Reserve, Orange Walk District, Belize, where bat sampling took place in April-May of 2017. Subsites where mist nets and harp traps were placed are indicated as magenta circles.

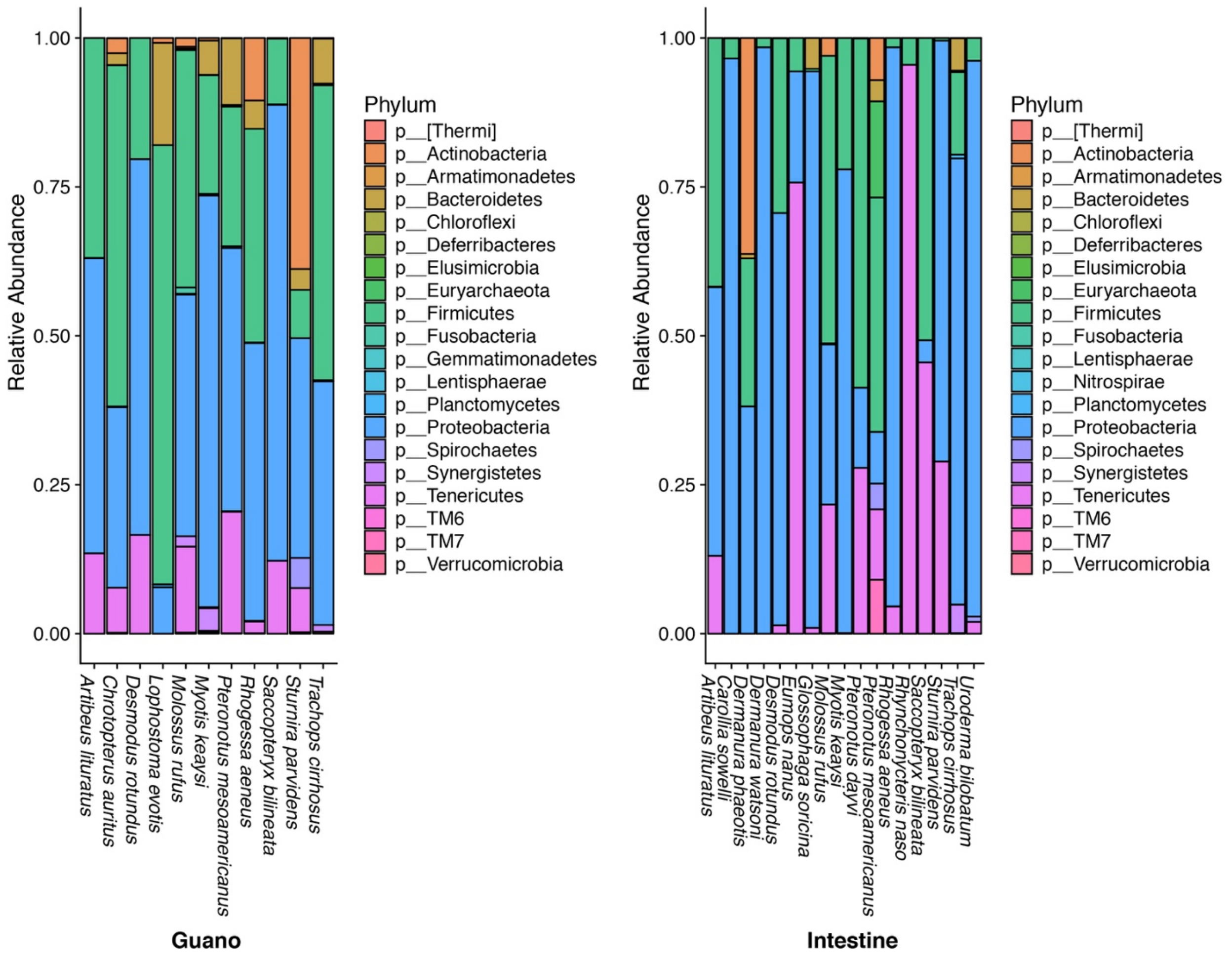

Supplementary Figure 2. Taxonomic bar plots depicting relative abundance of the top 20 most abundant bacterial phyla recovered in our analysis. On the left, guano samples are depicted; on the right, intestinal samples. Species with multiple biological replicates were aggregated to examine overall patterns in bacterial colonization across host species.

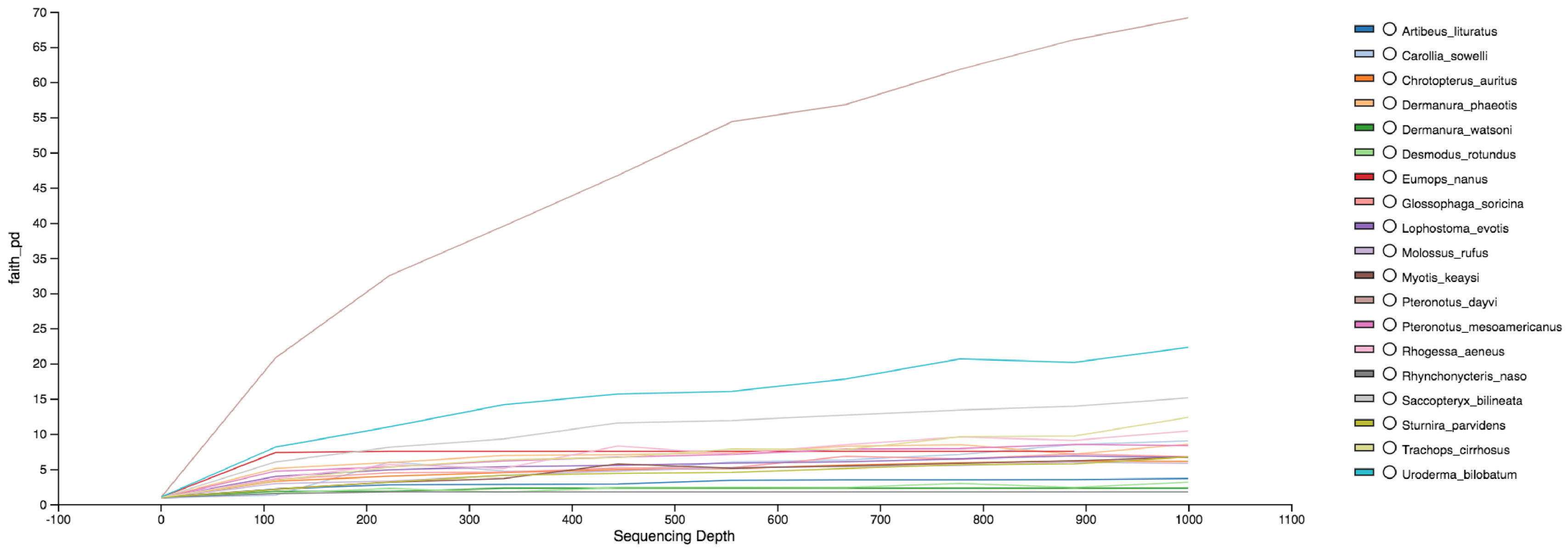

Supplementary Figure 3. Rarefaction curve for communities sampled in this analysis. Each colored line represents an individual bat, and each dot represents phylogenetic diversity (Faith's *PD*) computed for each sampling depth. Line labels to the right of the plot indicate the species corresponding to each curve.

A)

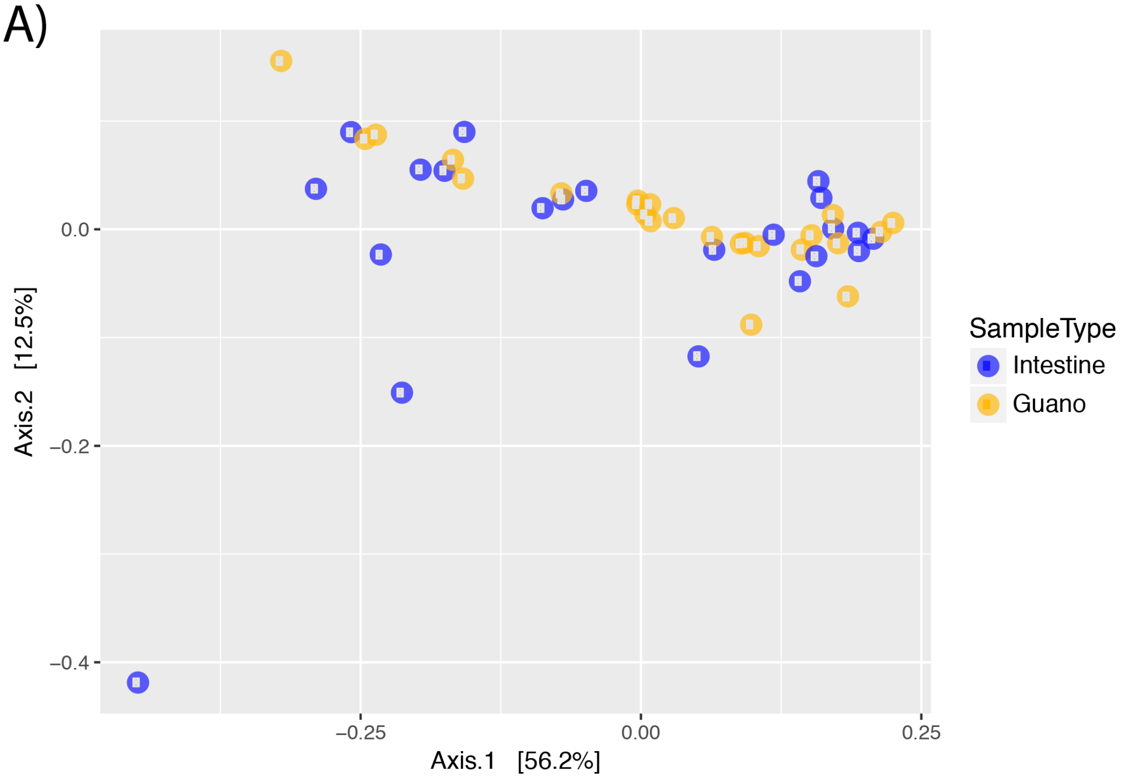

Axis.1 [56.2%]
Axis.2 [12.5%]
−0.25
0.00
0.25
0.0
−0.2
−0.4
SampleType
Intestine
Guano

B)

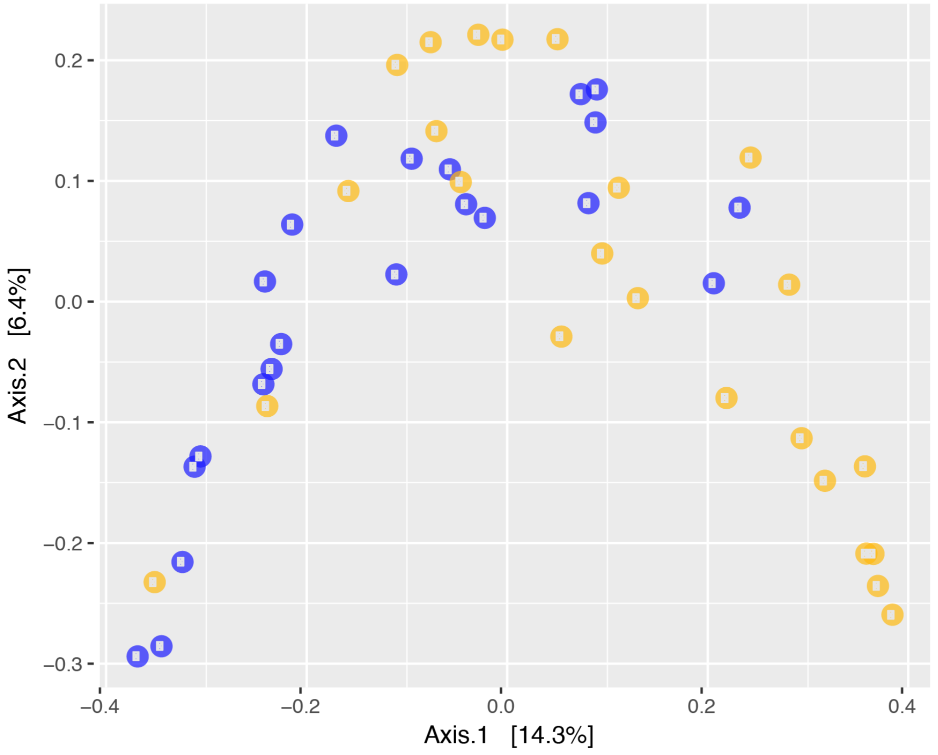

Axis.1 [14.3%]
Axis.2 [6.4%]
−0.4
−0.2
0.0
0.2
0.4
0.2
0.1
0.0
−0.1
−0.2
−0.3

Supplementary Figure 4. PCoA plots of all guano and colon microbiomes based on **(A)** weighted and **(B)** unweighted Unifrac distances. Weighted Unifrac distances were not significantly different between sample types (P = 0.243, F = 1.24, r^2 = 0.028). Unweighted Unifrac distances differed between sample types (P = 0.001, F = 3.35, r^2 = 0.072), but the effect size was small, and a test of homogeneity of dispersion was significant (P = 0.024, F = 5.94, Perm = 999), suggesting that differences in dispersion rather than difference in centroid position was driving this pattern.
